# The Role of Prestroke Glycemic Control on Severity and Outcome of Acute Ischemic Stroke

**DOI:** 10.1155/2014/694569

**Published:** 2014-09-08

**Authors:** Clara Hjalmarsson, Karin Manhem, Lena Bokemark, Björn Andersson

**Affiliations:** ^1^The Department of Cardiology, Sahlgrenska University Hospital, Blå Stråket 3, 1st Floor, 405 30 Göteborg, Sweden; ^2^The Stroke Unit, Department of Internal Medicine, Sahlgrenska University Hospital, Blå Stråket 5, 1st Floor, 405 30 Göteborg, Sweden; ^3^The University of Gothenburg, 405 30 Göteborg, Sweden

## Abstract

*Background/Aim*. Relatively few studies have investigated the association of prestroke glycemic control and clinical outcome in acute ischemic stroke (IS) patients, regardless of presence of diabetes mellitus (DM). The aim of this study was to investigate the importance of prestroke glycemic control on survival, stroke severity, and functional outcome of patients with acute IS. *Methods*. We performed a retrospective survival analysis of 501 patients with IS admitted to Sahlgrenska University Hospital from February 15, 2005, through May 31, 2009. The outcomes of interest were acute and long-term survival; the stroke severity (NIHSS) and the functional outcome, mRS, at 12 months. *Results*. HbA1c was a good predictor of acute (HR 1.45; CI, 1.09 to 1.93, *P* = 0.011) and long-term mortality (HR 1.29; CI 1.03 to 1.62; *P* = 0.029). Furthermore, HbA1c >6% was significantly correlated with acute stroke severity (OR 1.29; CI 1.01 to 1.67; *P* = 0.042) and predicted worse functional outcome at 12 months (OR 2.68; CI 1.14 to 6.03; *P* = 0.024). *Conclusions*. Our study suggests that poor glycemic control (baseline HbA1c) prior to IS is an independent risk factor for poor survival and a marker for increased stroke severity and unfavorable long-term functional outcome.

## 1. Introduction

Hyperglycemia (HG) in relation to acute IS is common both in patients with and in patients without a diagnosis of DM, and it has been suggested to worsen* survival*. However, recent results from several clinical studies indicate that particularly patients with stroke and stress HG, but not diabetes, have increased mortality [1–3].

On the contrary, older data by Woo et al. [4] found that patients with acute IS and similar glucose concentrations had similar outcome regardless of whether they had diabetes or not.

According to a review published by Capes et al. [5], acute HG predicted increased risk of in-hospital mortality after ischemic stroke (IS) in nondiabetic patients and increased risk of poor functional recovery in nondiabetic stroke survivors. The recent results of Nardi et al. [2] are also in line with this conclusion.

In a study published in 2012, Hu et al. [1] evaluated the effects of HG and prestroke glycemic control, as measured by HbA1c, on all-cause and cardiovascular mortality among 1277 IS patients and found a significant association between initial glucose level and mortality in nondiabetic patients. Surprisingly, they also found that DM patients with HbA1c <7.0% had a higher incidence rate of all-cause and cardiovascular death than those with HbA1c ≥7%. Contradictory data have been published by Kamouchi et al. in 2011, showing that patients with acute IS and DM had more unfavorable outcome, the higher HbA1c they had [6].

It is worth pointing out that several of the above-mentioned studies compared the patients by the clinical prestroke DM diagnosis, while no evaluation of HbA1c in the whole study cohort was available. As a result, there could have been interference from patients with subclinical DM who got included in the group without a diagnosis of DM.

A recent study by Li et al. [7] is one of the few studies systematically investigating the role of HbA1c on stroke outcome, regardless of a prestroke diagnosis of DM; their results show that elevated HbA1c level relates to stroke severity and poor prognosis in the whole study population; however, only patients with brainstem infarction were included in this study.

The primary aim of this study was to investigate the effect of prestroke glycemic control (glycated hemoglobin, HbA1c) on acute (30 days) and long-term (one year) survival of IS patients who did not undergo thrombolysis. The secondary aim was to investigate the effects of HG and HbA1c on acute stroke severity and on twelve-month functional outcome.

## 2. Methods

### 2.1. Study Design and Population

Epidemiological and clinical data, as well as laboratory parameters, were prospectively collected from 799 consecutive patients with acute ischemic stroke admitted to Sahlgrenska University Hospital from February 15, 2005, through May 31, 2009. Patients with transient ischemic attack and those with intracerebral hemorrhage were excluded. Also, patients in which HbA1c was not measured on admission (*n* = 298) were excluded. None of the patients included in the study received thrombolytic therapy. The local ethics committee approved the study.

### 2.2. Data Collection

Epidemiological data were obtained directly from patients, using standardized data collection instruments. When the patient was unable to provide answers, a proxy with knowledge of the subject's history was interviewed.

ECG, CT-scans, and blood tests were carried out on admission. Standard techniques were used to measure blood pressure, glycated hemoglobin, creatinine, fasting blood glucose, and serum lipid levels.

Patients were classified into two groups (DM, non-DM) according to the presence of a prestroke DM diagnosis. Hypertension was defined as systolic blood pressure >140 mm Hg or diastolic blood pressure >90 mm Hg (based on the average of two blood pressure measurements during hospital stay) or a patient's self-report of a history of hypertension or antihypertensive drug use. Diabetes mellitus was defined as the patient's self-report of such a history or use of insulin or a hypoglycaemic drug. The HbA1c data were used either as continuous variable or as dichotomous variable (HbA1c ≤6.0; >6.0, according to [8, 9]).

The diagnosis of stroke was assessed according to the World Health Organization definition of stroke [10]. The National Institutes of Health Stroke Scale (NIHSS) was used to assess the severity of the stroke [11].

Based on clinical and neuroimaging findings, stroke subtypes were classified according to the TOAST classification [12].

The modified Rankin Scale (mRS), validated for stroke outcome assessment, was used by a trained physician for assessing the functional outcome at 12 months after stroke. Favourable outcome was defined as mRS scores 0–2, corresponding to independence with regard to activities of daily living.

### 2.3. Outcomes

The primary end points of the study were the survival during the acute phase (up to 30 days after admission) and the long-term survival (up to 12 months after stroke debut).

Other outcomes of interest (secondary end points) were the initial stroke severity defined according to the NIHSS, used as continuous or dichotomized (NIHSS <7 and ≥7) variable, as appropriate, and the functional outcome, mRS, at 12 months, dichotomized as mRS ≤2 and >2.

### 2.4. Statistical Analysis

We used the Mann-Whitney *U* test for comparing the mean values of continuous variables (NIHSS, serum lipid levels, blood glucose, and blood pressure) by the DM group, while the chi-square test was used for discrete variables.

The associations between glycated hemoglobin on admission and stroke severity (number of points according to NIHSS, dichotomous NIHSS <7 and ≥7), as well as functional outcome at 12 months (dichotomous, mRS ≤2 and >2), were investigated using a logistic regression model, adjusting for age, gender, stroke subtype (TOAST criteria), admission blood glucose, previous TIA/stroke, and associated cardiovascular pathology. The same regression model was used for testing the relation between the above-mentioned outcome variables (NIHSS; mRS) and fasting blood glucose (continuous variable) on admission to hospital.

Survival was investigated using the Cox proportional hazard regression, where age, gender, stroke severity (as a continuous variable), stroke subtype, blood glucose, and prestroke medical conditions (previous TIA/stroke, previous myocardial infarction, atrial fibrillation, and DM) were used as covariates.

The log rank test (Mantel-Cox) was used to test the difference in equality distribution for the Kaplan-Meier curves. The results are presented as the hazard or odds ratio (HR, OR) with 95% confidence intervals (CI). *P* values <0.05 were regarded as statistically significant (2-sided test). All statistical analyses were performed using the SPSS 17.0 package (SPSS Inc., Chicago, IL, USA).

## 3. Results

### 3.1. Baseline Characteristics

The final dataset consisted of 501 patients. The mean age of the patients included in the analysis was 78.8 years and 48.5% were men.  Table 1 summarizes the demographics and baseline clinical characteristics of our study population in relation to prestroke diagnosed DM; we found 97 patients with DM; of these, 72 patients had type 2 DM; 68 patients were treated with per oral antidiabetics and 31 with insulin. No difference in stroke etiology was found between the DM versus the non-DM group.

There was no difference in mean systolic and diastolic blood pressure on admission between the two groups. However, the patients with DM were significantly younger (*P* = 0.013) and more often had prestroke hyperlipidemia (*P* < 0.001) and prestroke myocardial infarction (*P* = 0.024). The DM patients had significantly higher glycemia, HbA1c, total s-cholesterol, and s-triglycerides on admission; for details see Table 1.

### 3.2. Hyperglycemia

Three hundred ninety patients had glycemia above 7 mmol/L on admission; of these, 87 patients had known prestroke DM. The mean blood glucose level on admission to hospital was significantly higher (*P* < 0.001) in the patients with DM compared with the non-DM patients; for details see Table 1.

### 3.3. Glycated Hemoglobin (HbA1c)

In the entire study population, 13.2% had HbA1c >6% (min 3.1%, max 14.9%) on admission to hospital. About 50% of the patients with diabetes had HbA1c >6.0%, while a total of seventeen patients with HbA1c >6.0% were identified among those without a previously known diagnosis of DM.

### 3.4. Primary End Points: Acute and Long-Term Survival

#### 3.4.1. Acute Survival

**Figure 1:**
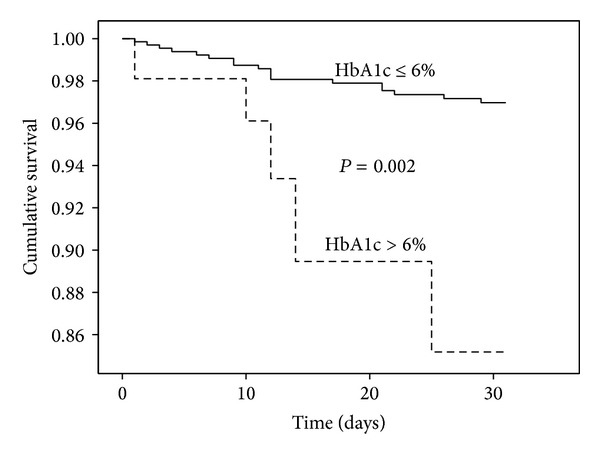
Cumulative survival during the first month after acute ischemic stroke (*n* = 39 deaths); stratification according to level of glycated haemoglobin, HbA1c.

The 30-day all-cause mortality rate was 7.8% (*n* = 39). In a survival analysis by Cox proportional hazard model, the level of HbA1c, used as continuous variable, was a strong predictor of acute mortality (HR 1.45; CI, 1.09 to 1.93, *P* = 0.011), after adjusting for relevant covariates: age, stroke severity, stroke subtype, atrial fibrillation, previously diagnosed DM, blood glucose, and previous TIA/stroke. Moreover, HbA1c >6% was also a robust predictor of increased mortality (HR 6.72; CI, 2.02 to 22.34, *P* = 0.002); for details see Figure 1 and Table 2.

Glycemia on admission influenced the acute survival in a significant manner (HR 1.16; CI, 1.02 to 1.32; *P* = 0.028), independent of age, stroke severity, previous TIA/stroke, previously diagnosed DM, and atrial fibrillation. However, when adjusting for prestroke glycemic control (HbA1c), hyperglycemia was no longer an independent predictor.

#### 3.4.2. 12-Month Survival

**Figure 2:**
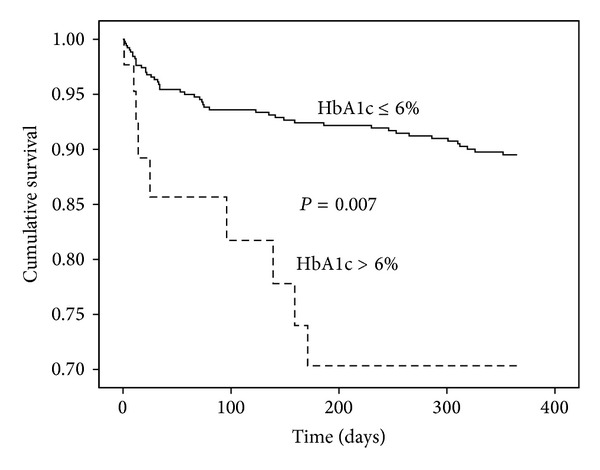
Cumulative survival during the 12-month followup after admission to hospital (*n* = 98 deaths); stratification according to level of glycated haemoglobin, HbA1c.

Ninety-eight deaths were registered during the first year after stroke (cumulative mortality rate 19.6%). The level of HbA1c (HR 1.29; CI 1.03 to 1.62; *P* = 0.029) on admission, used as continuous variable, was an independent predictor of 12-month survival after adjusting for the relevant covariates mentioned above. Also, when dichotomized, HbA1c >6% was significantly associated with worse survival (HR 3.40; CI 1.40 to 8.22; *P* = 0.007); for details see Figure 2 and Table 2. Glycemia was associated with long-term survival (HR 1.12; CI 1.01 to 1.24; *P* = 0.031) but lost its predictive value when the analysis was adjusted for the prestroke glycemic control (HbA1c).

### 3.5. Secondary End Points: Stroke Severity and Functional Outcome

#### 3.5.1. Stroke Severity

In an age-, gender-, stroke-, subtype-, and comorbidity-adjusted linear logistic regression, HbA1c, but not glycemia, was significantly correlated with acute stroke severity (NIHSS ≥7; OR 1.29; CI 1.01 to 1.67; *P* = 0.042); other significant predictors of stroke severity were age (*P* = 0.006), gender (*P* = 0.026, lower risk for men), atrial fibrillation (*P* < 0.001), and prestroke DM (*P* = 0.044).

#### 3.5.2. Functional Outcome

The HbA1c >6% predicted worse functional outcome (mRS > 2) at 12 months after stroke (OR 2.68; CI 1.14 to 6.03; *P* = 0.024) in a binary regression analysis adjusted for age (OR 1.07; CI 1.04 to 1.12; *P* < 0.001), previous stroke/TIA (OR1.99; CI 1.09 to 3.62; *P* = 0.025), and stroke severity (OR 1.24; CI 1.17 to 1.31; *P* < 0.001); gender, stroke subtype, and glycemia on admission were not related to functional outcome, as defined above.

## 4. Discussion

This is one of the few clinical studies where the role of glycated hemoglobin is systematically evaluated with respect to early and late outcome after IS regardless of whether patients had DM or not. Our results demonstrate that poor prestroke glycemic control is (1) an independent determinant of stroke severity, (2) a good predictor of acute and long-term survival, and (3) a robust marker of neurological functional outcome.

A large percentage of our patients (77.8%) had HG on admission, but only 19.4% had prestroke DM; thus, “stress HG” in the absence of DM is very common.

Several previous studies have found worse survival in patients with HG [1, 2, 4, 5, 13]. Some research groups [1, 2, 5] have reported that HG is a determinant of worse clinical outcome in patients with IS without DM, but not in those with DM, while others [13] have found a deleterious effect of HG on survival regardless whether or not the patients had DM. In our study, HG was related to increased mortality, but it lost its predictive value when the analysis was adjusted for prestroke glycemic control. Also, HG was not correlated with stroke severity in an independent manner, even though it has preliminarily been linked to induction of procoagulant state and to neurotoxicity, due to prooxidative and proinflammatory effects [14]. HG did not influence the functional outcome one year after stroke either.

In this work, we found a strong relationship between prestroke glycemic control and neurological outcome, as well as survival. HbA1c was a good independent predictor of stroke severity in the whole study group, not only in patients with DM. Moreover, we observed a fine incremental effect, testing the HbA1c as a continuous variable as well, not only as a categorical one.

Results from two newly published studies [15, 16] also show that high HbA1c is independently associated with poor outcome 1 year after stroke, supporting our findings. Nevertheless, one of the studies, based on data from the Fukuoka Stroke Registry [15], only included patients with known DM; moreover, none of the studies investigated the effect of HbA1c on acute stroke severity.

The exact mechanism by which poor prestroke glycemic control affects survival of stroke patients is less clear; general complications related to poorly controlled DM could be one explanation. An increased HbA1c level reflects poor long-term glycemic control and has specific implications for the structure and function of the vascular bed, including small and large cerebral vessels. Increased HbA1c level might also be a marker of poor compliance, indicating an unhealthy lifestyle.

In our study, no relationship between HbA1c and the type of cerebrovascular lesion, as defined by TOAST criteria, was observed (data not shown). This finding is in line with the results of Heo et al., [17] who did not find any differences in the incidence of large artery disease, leukoaraiosis, cerebral microbleeds, and/or old lacunes by the level of HbA1c in patients with acute ischemic stroke. Thus, even though one would expect patients with diabetes to be more prone to developing stroke secondary to large artery disease and/or small vessel disease, no differences were noted in this study group. Of course, an explanation could be that our study population is quite old and, therefore, has a pronounced cardiac comorbidity, which may even out the potential differences.

One of the limitations of this study is that we did not measure the size of the stroke lesion by CT or MRI; however, it is well known that the NIHSS score is a good clinical outcome measure, which parallels infarct volume [18].

Another limitation is related to the lack of data on the duration of diabetes and serial measurements of the HbA1c during the follow-up period.

In summary, our study demonstrates that impaired prestroke glycemic control (baseline HbA1c) is an independent risk factor for poor survival and for unfavorable functional outcome after IS. Some relatively recent randomized controlled trials [19–21] have found that intensive glycemic control could not reduce cardiovascular risk in diabetic patients, but it is important to highlight that these studies used a cutoff HbA1c higher than 6.4%. Therefore, additional studies are needed to elucidate whether intensified prestroke glycemic control may improve clinical course and outcome in patients with acute ischemic stroke.

## Figures and Tables

**Table 1:** Patients' characteristics at baseline (*N* = 501) by presence of diabetes mellitus.

Baseline characteristics	No diabetes mellitus (*n* = 404)	Diabetes mellitus (*n* = 97)	*P* value
	*N* (%)	*N* (%)	
Gender, female	219 (54.2)	39 (40.2)	0.013∗
Age, years (SD)	79.2 (7.8)	76.5 (8.8)	0.003^∗∗a^
Hypertension	231 (57.2)	64 (66.0)	0.114
Hyperlipidemia	52 (12.9)	26 (26.8)	0.001∗∗∗
Atrial fibrillation	139 (34.4)	29 (29.9)	0.398
Heart failure	48 (11.9)	15 (15.5)	0.344
Previous myocardial infarction	51 (12.7)	21 (21.6)	0.024∗
Previous intracerebral hemorrhage	7 (1.7)	1 (1.0)	0.617
Previous ischemic stroke	99 (24.5)	32 (33.0)	0.088
Previous TIA	26 (6.4)	10 (10.3)	0.187
Stroke subtype (TOAST)			
Atherothrombotic	92 (22.7)	31 (31.9)	0.053
Cardioembolic	151 (37.4)	28 (28.9)	0.127
Small vessel disease	109 (27.0)	23 (23.7)	0.537
Other causes	3 (0.7)	0	0.396
Unknown cause	48 (11.9)	14 (14.4)	0.476
Assessments, mean (SD)			
Plasma glucose, mean (SD), mmol/L	7.1 (1.8)	10.2 (3.9)	<0.001^∗∗∗a^
HbA1c (SD), %	4.9 (0.7)	6.5 (1.6)	<0.001^∗∗∗a^
Systolic blood pressure, mean (SD), mmHg	166.8 (31.1)	160.6 (25.7)	0.079^a^
Diastolic blood pressure, mean (SD), mmHg	92.7 (16.5)	86.0 (17.2)	0.001^∗∗∗a^
S-cholesterol, total (SD), mmol/L	4.3 (1.1)	5.0 (1.2)	<0.001^∗∗∗a^
S-LDL (SD), mmol/L	3.1 (1.0)	2.8 (2.5)	0.097^a^
S-HDL (SD), mmol/L	1.5 (0.5)	1.3 (0.5)	<0.001^∗∗∗a^
S-TG (SD), mmol/L	1.2 (0.6)	1.5 (0.8)	<0.001^∗∗∗a^
NIHSS, mean (SD)	6.5 (6.8)	5.8 (7.0)	0.391^a^
NIHSS > 7	109 (26.9)	19 (19.6)	0.094

*P* values, according to chi-square test.

^a^Mann-Whitney *U* test.

*P < 0.05; **P < 0.01; ***P < 0.001.

**Table 2:** Multiple logistic regression analysis showing the relationship between survival and explanatory variables.

Explanatory variable	30 days after stroke			One year after stroke		
	HR∗	95% CI	*P *value	HR∗	95%CI	*P *value
Age (years)	1.04	0.96–1.12	0.321	1.05	1.01–1.09	*0.037 *
Gender, female	0.70	0.31–1.62	0.407	0.71	0.40–1.24	0.223
Diabetes mellitus	6.15	1.15–32.88	*0.034 *	3.06	1.07–8.73	*0.037 *
Fasting blood glucose	1.12	0.89–1.41	0.344	1.08	0.94–1.25	0.273
HbA1c	6.72	2.02–22.34	*0.002 *	3.40	1.40–8.22	*0.007 *
Atrial fibrillation	1.20	0.48–3.02	0.698	1.21	0.68–2.16	0.525
Previous myocardial infarction	0.39	0.09–1.69	0.206	1.16	0.57–2.34	0.681
Previous TIA/ischemic stroke	2.24	0.95–5.31	0.067	1.55	0.87–2.75	0.134
Stroke severity (NIHSS)	1.15	1.10–1.20	*<0.001 *	1.10	1.07–1.13	*<0.001 *

*HR for increase of age by one year or presence of a specific medical condition.

HR <1 indicates factor favours survival; HR >1 indicates factor is associated with poor survival.
